# Making doctors stay: Rethinking doctor retention policy in a contracted-out primary healthcare setting in urban Bangladesh

**DOI:** 10.1371/journal.pone.0262358

**Published:** 2022-01-05

**Authors:** Farzana Bashar, Rubana Islam, Shaan Muberra Khan, Shahed Hossain, Adel A. S. Sikder, Sifat Shahana Yusuf, Alayne M. Adams

**Affiliations:** 1 Health Systems and Population Studies Division, International Centre for Diarrheal Disease Research, Bangladesh (icddr,b), Dhaka, Bangladesh; 2 School of Population Health, University of New South Wales, Sydney, New South Wales, Australia; 3 Infectious Disease Division, International Centre for Diarrheal Disease Research, Bangladesh (icddr,b), Dhaka, Bangladesh; 4 Department of Family Medicine, Faculty of Medicine and Health Sciences, McGill University, Montreal, Quebec, Canada; Universiti Pertahanan Nasional Malaysia, MALAYSIA

## Abstract

**Background:**

*“Contracting Out”* is a popular strategy to expand coverage and utilization of health services. Bangladesh began contracting out primary healthcare services to NGOs in urban areas through the Urban Primary Health Care Project (UPHCP) in 1998. Over the three phases of this project, retention of trained and skilled human resources, especially doctors, proved to be an intractable challenge. This paper highlights the issues influencing doctor’s retention both in managerial as well as service provision level in the contracted-out setting.

**Methodology:**

In this qualitative study, 42 Key Informant Interviews were undertaken with individuals involved with UPHCP in various levels including relevant ministries, project personnel representing the City Corporations and municipalities, NGO managers and doctors. Verbatim transcripts were coded in ATLAS.ti and analyzed using the thematic analysis. Document review was done for data triangulation.

**Results:**

The most cited problem was a low salary structure in contrast to public sector pay scale followed by a dearth of other financial incentives such as performance-based incentives, provident funds and gratuities. Lack of career ladder, for those in both managerial and service delivery roles, was also identified as a factor hindering staff retention. Other disincentives included inadequate opportunities for training to improve clinical skills, ineffective staffing arrangements, security issues during night shifts, abuse from community members in the context of critical patient management, and lack of job security after project completion.

**Conclusions:**

An adequate, efficient and dedicated health workforce is a pre-requisite for quality service provision and patient utilization of these services. Improved career development opportunities, the provision of salaries and incentives, and a safer working environment are necessary actions to retain and motivate those serving in managerial and service delivery positions in contracting out arrangements.

## Introduction

Human Resources for Health (HRH) is an essential pillar to achieve Universal Health Coverage (UHC) [1] and a crucial “building block” in national health systems [2]. As quality care and patient satisfaction largely depend on skilled HRH, lack of experienced health professionals adversely affects the quality and quantity of the healthcare services, and undermines progress towards UHC [3]. The need to strengthen the health workforce is an almost universal agenda [4]. Low, middle and even high income countries report difficulties in recruiting and retaining experienced and qualified doctors [5–8]. According to WHO, in 2006 there was a global shortage of 4.3 million healthcare workers [9] which is particularly acute in rural remote and hard-to-reach areas [8, 10, 11]. Also ubiquitous is the high concentration of doctors in urban areas and related efforts to reallocate healthcare workers to enable a more equitable and appropriate skill mix across rural and urban areas [8, 12, 13].

In Bangladesh, a closer examination of the urban health system reveals another layer of inequities in HRH distribution that privileges specialist care and the private sector. The skilled HRH shortage is particularly acute at the primary care level where no publicly organized system is in place [12, 14–16]. Not only the HRH shortage, due to several other factors including education of the parents, financial condition, housing, water and sanitation system, inequities abound specially when it comes to slum areas [17, 18]. According to the recent estimates, the national average of 4+ Antenatal Care (ANC) coverage is 37% (48.8% urban and 33.1% rural) [19]. But disaggregation within urban areas reveals a two-fold difference in 4+ ANC between urban slum (66.4%) and urban non-slum (32.9%) [20]. Again, in urban slums, the prevalence of stunting is 1.53 times higher than non-slum areas [17]. Such urban/rural or slum/non-slum disparities are longstanding and Government of Bangladesh recognized it decades ago. To bridge this gap, Bangladesh’s Ministry of Local Government, Rural Development and Cooperatives (MOLGRD&C), under whose mandate urban primary healthcare falls, has contracted-out Non-Governmental Organizations (NGOs) to meet the primary care need of the urban poor under the Urban Primary Health Care Project (UPHCP) [21, 22]. Initiated in 1998, the original aim of the Contracting-Out (CO) process was to serve the urban poor as well as to gradually build capacity of the local government bodies (e.g. municipalities, city corporations) to supply and manage services themselves [22]. A variety of actors participated in the CO process including donors, the Government of Bangladesh, NGOs and other collaborating partners which changed over the three phases of the project: Phase 1 (1998–2005), Phase 2 (2005–2012) and Phase 3 (2012–2018). Presently, the project’s fourth phase (2018–2023) is ongoing [23]. A separate body named the Project Management Unit (PMU) was formed to oversee the CO process and its monitoring and management. Under the PMU, the implementing agencies of the project were the respective City Corporations (CCs) and municipalities working through a Project Implementation Unit (PIU). Partner NGOs were responsible for managing clinical service provision [24, 25].

Though this CO model brought much needed primary care services to otherwise underserved urban dwellers, various implementation challenges were experienced, one of which was the rapid turnover of doctors [26, 27]. Project documents revealed several issues around human resources including poor staff retention, partly due to reductions in incentive mechanisms and capacity building opportunities [25, 28, 29]. In this paper, we analyzed the key challenges to doctor recruitment and retention in the first three phases of the project, with a view to identifying how these might be overcome. To create a more responsive and efficient health workforce, it is imperative to understand labor market dynamics as well as project-related factors which influence doctor retention. Insights from this analysis will be useful in guiding future CO efforts in similar low- and middle-income urban settings with weak public systems.

## Methods

### Study setting and period

In rural Bangladesh, the Ministry of Health and Family Welfare (MOH&FW) provides free access to basic primary health care services, reproductive and family planning services through a well-structured system of community clinics, and sub-district and district level hospitals, albeit subject to widespread human resource shortages at every level [12, 30]. By contrast, the urban health system is governed jointly by two ministries: the MOH&FW and the MOLGRD&C [18]. The MOH&FW is responsible for healthcare provision in urban secondary and tertiary hospitals, many of which are overstretched due to patient load [25]. Urban Primary Health Care provision falls under the purview of local municipal government under the MOLGRD&C [31], but remains largely undeveloped due to inadequate human and financial resources [25, 31]. This bifurcated system of health governance in urban areas has served to complicate healthcare provision pathways and resulted in the growth of the private sector in health [18, 32]. Concerns about quality of care and equitable access of private sector services, and weak regulatory systems are the focus of the latest national strategic plan of Bangladesh [33–35]. One way of engaging with the private sector in expanding health services coverage in urban areas is through contracting-out arrangements. The UPHCP is one such example and the subject of this paper.

This paper focuses on HRH issues and is based on data from a larger study. The larger study aimed to explore the evolution of the CO process over the UPHCP’s first three phases in Bangladesh and was published elsewhere [36]. Forty-two key informant interviews (KIIs) were conducted with donors, officials from the MOH&FW, UPHCP personnel and NGO representatives. Data collection was conducted from November 2015 to April, 2017 (Table 1).

**Table 1:** Distribution of study respondents.

Respondents	Phase wise KI distribution				Main issues explored
	1^st^	2^nd^	3^rd^	Total	
Donors (*DNR*)	1	4	3	8	Overall all implementation procedure of the project, Role of MOH&FW, MOLGRD&C Input and output management, Service delivery approach, Barriers and challenges of health workforce
Contract Designer (*CDG*)	1	2	1	4	
Ministry of Health and Family Welfare *(GoB)*	3	1	-	4	
Project Administration (PMU, PIU) (*PRL*)	5	7	8	20	
NGO Head/ NGO Manager (*NGM*)	7	8	6	21	
Clinic Medical Officers (*CMO*)	-	-	7	7	
**Total**	**17**	**22**	**25**	**64** *	

*Key informants involved in multiple phases were counted more than once. Hence, number of interviews was 64 instead of 42.

### Study design

The study employed an exploratory qualitative approach to data collection, supplemented by desk review. Tailored, open ended Key Informant Interview (KII) guidelines were developed for different categories of key informants based on two theoretical frameworks, the Health Policy Triangle by Walt & Gibson (1994) [37] and Liu et al.’s (2004) framework for monitoring and evaluating primary healthcare contracting-out interventions [38]. All the interview guidelines were field tested before initiating formal data collection.

### Sampling method and techniques

Study participants were selected both purposively and using a snowball sampling approach. KIIs were conducted with individuals who were involved in any of the UPHCP’s three different phases for at least six months. Direct service providers (medical officers) from NGOs (from third phase only) were also interviewed. NGO managers and doctors were purposefully selected from NGOs both inside and outside Dhaka (Capital of Bangladesh) and from low, medium and high performing NGOs (categorization based on project evaluation reports) to capture and ensure maximum variation of data. Our efforts to capture diverse perspectives at different levels and locations helped to generate a reliable, valid and comprehensive dataset by allowing contrast and comparison between respondents. The trustworthiness of data was further enhanced by conducting face to face interviews at the convenience of participants and by ensuring confidentiality and anonymity.

### Data collection techniques

Qualitative interviews lasted between 30 to 90 minutes. Some interviews were audio-recorded with permission of the KIs; if permission to record was denied, detailed notes were taken. After 42 interviews, data saturation was reached. Desk review involved an extensive scoping of relevant policy documents, like project proposals, contract agreements, project completion reports, donor reports, program log frames, and Quarterly Performance Reports. Notes were reviewed by two researchers conducting the interview to ensure all important information was captured accurately. All the interviewers (five females and two males) were full-time researchers with extensive experience in health systems research and conducting qualitative interviews.

### Data analysis

Prior to data collection, an *a priori* code book was generated (for both interviews and document review) based on the two frameworks. Code definitions were revised and refined throughout the data collection process. Immediately after completion of each interview, digital recordings or hand-written notes were transcribed (in Bengali) and translated into English. Team-based coding was performed using the software ATLAS.ti (version 7). Data displays were created to structure the analysis process and to identify patterns and themes across respondents and CO phases. Data analysis and interpretation were also informed by a consultative workshop with key stakeholders and several one-to-one meetings with KIs. Their feedback and reviews helped sharpen and validate our conclusions and recommendations. Further details on methodology are available in a prior publication reporting on the larger study [36].

### Ethical considerations

Ethical approval for the study (PR-15088) was received from the Institutional Review Board of icddr,b. Prior to interview, KIs were asked to provide informed written consent after having reviewed the study’s objectives, procedures, and measures to protect anonymity and confidentiality. All respondents were provided with unique identifier codes to maintain anonymity. Any identifier (i.e. name, place of interview, working organizations etc.) in the transcripts were also de-identified to preserve confidentiality.

## Results

A number of barriers and challenges were faced by doctors in managerial and provider roles which relate to poor retention and compromised service quality.

### Low and variable salaries and benefits

Across all three phases of the project, the most frequently reported barrier to doctors’ retention was the low payment scale relative to the Government’s salary structure. Doctors working in public sector jobs were reported to earn twice that of doctors engaged in the project:

“*Our salary is too poor*, *BDT 21*,*767 per month (approx*. *270 USD)*. *We have not received a salary increase even after 1*.*5 years*… *This is my last month here*. *I will attend my exam [MD] soon*. *After that*, *I will try to join (as a medical officer for) the Bangladesh Army*.*” (Phase 3*: *CMO-07)*

Low wages were partly a consequence of the way in which contract bidding criteria and processes were structured. Due to the Asian Development Bank’s (ADB) procurement guidelines, the bidding process to select NGOs underwent a major change from Phase 2 to 3. In previous phases, the proposals were evaluated in two steps. First, they were technically assessed, and then only technically sound proposals were considered for financial assessment. The contract was awarded to NGOs with the highest combined score (both technical and financial). However, in the third phase these two procedures were merged. Priority was given to the financial part over technical part, and the lowest bidder was awarded the contract–essentially, a “low-cost bidding system” was adopted. This made it almost impossible to increase levels of remuneration later in the contract. Salaries, as a result, varied across partnership areas with a minimum wage (ceiling). Furthermore, during transition periods between phases, delays in funding occurred resulting in the temporary cessation of salary for all employees including doctors. All these factors negatively impacted doctors’ retention in the project:

“*Among those who were qualified*, *if they got a better offer*, *they switched to another job*. *Staff retention was very bad and turnover was high*.*” (Phase 1*: *PRL-01)*

Further diminishing doctor retention was a lack of consistent salary supplements (e.g., provident fund, gratuity, or performance bonus). In the first two phases, doctors were offered options for a provident fund and gratuity acknowledging their service, as well as festive bonuses. In the third phase, project staff received only salary and festive bonuses. As one clinic manager explained:

“*It is difficult to retain trained staff*… *Because*, *this [is an] NGO job*, *there are no benefits like a Government job*, *there is neither allowance nor benefits*, *nothing*.*” (Phase 1 and 2*: *NGM-05)*.

While funds were reserved for performance bonuses across all phases of the project, they were only disbursed in the first phase:

“*Several crore (1 crore = 10 million BDT) was kept [as a performance-based incentive for NGOs but the Project Management returned it to ADB (in Phase 2)… In the previous phase we got it… Each of us got 15*, *20*, *even 30 thousand taka (400 USD)… This [incentive]…was important [for staff retention]*.*” (Phase 1 and 2*: *NGM-01)*

### Absence of opportunities for career development

Another issue affecting doctor retention was the failure of project structure to accommodate the need for professional growth. Doctors working in the clinics did not have opportunities for career development or promotion as senior clinicians. As one Government of Bangladesh employee noted:

“*Doctors do not want to take posting here because this is a block post*, *so*, *he will not be promoted*. *His career will be hampered*. *That’s why they [the project authority] do not get (qualified) physicians*.*” (Phase 2*: *GOB-02)*

In addition to clinical duties, many doctors were obliged to work in managerial roles within the PIU at the CC or, municipality level, or as project managers in NGO clinics. These responsibilities were not perceived to add value to their clinical qualifications. Only in the four largest CCs there was a provision for career progression from Assistant Health Officer, to Health Officer, to Deputy Chief Health Officer. For most other CCs and municipalities, there was no opportunity for promotion or study leave to obtain an advanced level post-graduation degree. As one respondent explained:

“*There is no better opportunity*. *Suppose*, *one person has joined in ‘XX’ Municipality*… *The one and only post for this is called Health Officer*. *He has no scope for promotion or demotion*. *If he serves there for 30 years*, *he will serve in the same post*. *There is no career ladder*.*”(Phase 1*, *2 and 3*: *PRL-09)*

### Heavy workloads and no time off

Many respondents noted that medical officer burnout was a common phenomenon due to shortages of adequate skilled personnel. Usually, each Comprehensive Reproductive Health Care Centre (CRHCC) offering 24/7 services had four duty doctors on staff. One duty doctor was assigned to cover each eight-hour shift with the exception of the morning hours when consultants helped to manage a typically huge influx of patients. This workload was considered excessive by many doctors, affecting their ability to take leave during government holidays or weekends due to full duty rosters and staff shortages. One doctor noted that this also negatively affected opportunity for professional development:

“*During the service period*, *physicians get minimum training because as there is roster duty and [no extra physician]*, *so if any doctor go for a training then there is none to replace*.*” (Phase 3*: *CMO-06)*

### Inadequate training and skill development

In the initial phase of the project, doctors underwent lengthy training (six months to one year for Emergency Obstetric Care (EOC) or Anesthesia), but in the third phase, only short formal trainings (with duration of three to five days) were offered on EOC, menstrual regulation and family planning arranged by the PMU. These were perceived as inadequate by some doctors:

“*I have attended several training sessions*, *for example*, *on post-abortion care*, *tuberculosis*, *etc*. *But it is not sufficient to cover all the aspects” (Phase 3*: *CMO-02)*.

Due to the inadequacy of training, and the lack of specialized personnel or round-the-clock senior level physicians (obstetricians and pediatricians) to supervise the medical officers, several respondents noted that critical cases tended to be referred to nearby health facilities. Indeed, lack of opportunity for mentorship from senior role models was expressed as a major gap for junior medical officers seeking to acquire specialized skills in critical patient management:

“*In this post of medical officer*, *the rate of turnover is very high*. *Everyone has own ambition*, *to be specialized doctor*. *But*, *here*, *there is no opportunity to be specialized*…,*[and] towards the development of [clinical] skills…So*, *we [only] handle the general cases*. *But it does not serve the aim of a professional doctor” (Phase 3*: *CMO-02)*.

### Job insecurity

According to key informants, substantial job insecurity was experienced given that there was no guarantee that an NGO in charge of a specific partnership area would retain that area or even maintains a contract across phases of the project. New bids were required for each phase, and new contracts and partnership areas were established which may or may not require the full complement of staff in the prior phase. The absence of job security was considered one of the principal reasons for doctor turnover. As one NGO manager explained:

“*[not only] there was no salary [in the transition period]… it was also not guaranteed that our NGO would be selected for the third phase (Phase 3*: *CMO-01)*.

### Lack of workplace safety and dignity

Workplace-related safety concerns were expressed by several female participants and linked to unmet community expectations for specialized medical attention. As one doctor elaborated:

“*We do not admit [critical patients] newborn and children*, *for example*, *premature neonates*, *low birth weight*, *pneumonia*, *etc*. *because we do not have those kinds of [specialized] facilities in our CRHCC [health facility]*. *We refer them to Sadar [Tertiary/district] Hospital” (Phase 3*: *CMO-03)*

However, community members were not always aware of these limitations. In these circumstances, if a patient becomes critically ill or dies, anger and frustration were sometimes expressed in a manner that undermines the safety of staff. For female doctors, these concerns are particularly acute, especially during night shift duties when fewer staffs were present, and opportunities for abuse were more likely to occur. Disrespect was also shown to doctors during community meetings which were held periodically to ensure community participation. As a result, some doctors working in managerial posts were reluctant to interact with local political and community leaders and, were actively searching for a safer and more secure job environment.

### Managerial insensitivity to the needs of doctors

The tendency to appoint non-technical personnel into managerial roles in the project also led to discontent among doctors. For example, the Project Director in one of the phases had no prior experience of working in the health sector, while within PIUs, doctors were frequently working under non-physician Chief Executive Officers (CEOs). It was critical to convince the Project Director without a medical background about a health project’s need (i.e. specialized training for doctors) because they lacked technical expertise in health or healthcare management. One doctor shared that once a Project Director refused to approve training on comprehensive new-born care package, as he perceived project was focused on “primary healthcare”.

“*In a health project*, *non-technical persons are mentoring and supervising technical persons*. *It is difficult to make a non-technical person understand health service related activities*…*It is not good for a health project*.*” (Phase 3*: *PRL-10)*

Project personnel explained how non-physician Project Directors lacked understanding of medical education curriculum and pedagogy (i.e. theoretical sessions followed by practical classes), citing difficulties in persuading them about the importance of quality assurance and related capacity building for staff. One key informant narrated his experience:

“*When it ‘[training plan] was forwarded to Project Director*, *he asked the program officer and me*, *“Why is it [number of participants] 15*, *why not 30*?*” I explained*, *“Sir*, *this is an important training on RTI/STI [reproductive tract infections/sexually transmitted infections]*. *The trainees need to learn about the symptoms and treatment of patients… If the number of participants is 15*, *it becomes easier to following the steps of learning*, *that is*, *observing*, *assisting and acting (practicing)*. *Now*, *if I want to ensure quality training*, *I will select 15 participants*, *five participants per outdoor [sessions]*. *Now*, *if I send 30 participants*, *then it will be 10 participants per outdoor*. *No one will learn anything*. *Ultimately*, *nothing will happen*!*” (Phase 3*: *PRL-10)*

## Discussion

Drop-out and turnover of health professionals represent a significant economic burden to CO projects and systems that support them [39], resulting in loss of resources invested in training and education, and decreased quality of care and healthcare coverage [3, 40]. In Bangladesh, poor doctor retention contributes to the country’s HRH crisis and poses a key obstacle to achieving the goal of UHC [12]. In rural and remote areas, vacancy and absenteeism in the medical sector is wide spread [12, 16, 41], with almost 20% of total posts remaining vacant each year [35]. Of note, in this study, government CO primary care services in urban areas are also experiencing severe HRH shortages that compromise quality of care especially among poor and underserved populations.

Motivational factors behind doctor retention are undoubtedly context-specific but a recent systematic review identified three core considerations, among which financial issues are central [42]. Correspondingly, our study found that wage inequalities (vis a vis the public sector), and insufficient financial incentives, were key factors de-motivating CO doctors to remain in their posts. Over the three phases of the project, government sector salaries rose in fairly frequent intervals (twice in 2009 and 2015) [43, 44] to levels nearly double that of equally trained doctors working for CO NGOs, contributing to a “brain drain” of doctors hired by project NGOs. Similar effects of low salaries on doctor recruitment and retention in health sector reform arrangements have been noted in other studies conducted in Bangladesh [45], and other parts of the world [46, 47]. Although measures to rectify the problem were approved by ADB, a revised salary scale was not implemented until the third phase of the project due to bureaucratic complications. Interestingly, while the literature suggests that CO mechanisms can minimize bureaucratic inefficiencies associated with public sector provision, i.e. it usually involves six ministries to create a new HRH post, and a recruitment wait-time of two to three years to fill a vacant post in the public sector, our findings suggested otherwise [12, 48, 49]. Delays in fund disbursement and salary support occurred regularly especially in the initial phases of the project, and were further exacerbated during transition periods between CO contracts. Similar delays in disbursements of funds have been reported in previous donor-funded projects involving non-state providers [35, 50].

Numerous countries have adopted financial and performance-based incentives and other salary supplement policies to prevent the attrition of physicians [10, 51] by augmenting low base pay and improving quality of life when base pay is insufficient. Incentives have also been successfully employed to improve provider performance, service quality and health outcomes [52]. Even though the third phase of UPHCP had provision to offer performance-based incentives for qualified NGOs and their project staff, these were not actioned. Our study showed that financial incentives were initially accommodated, but insufficient and reduced phase-by-phase [25, 28, 29]. The low-cost bidding procedure introduced in Phase 3 to select partner NGOs resulted in a further erosion of incentives as cost reduction was prioritized over efforts to motivate the retention of skilled human resources, and NGOs focused on more on winning contracts versus improving service quality. This finding emphasizes the importance of bolstering managerial authority so that capacity to implement evidence-based strategies is strengthened, and donors or monitoring agencies have the authority to intervene if implementation processes are not followed.

Another key issue affecting doctor retention was failure to adopt measures that would increase doctor motivation such as opportunities for promotion and career development. In fact, over each phase of the project these opportunities were reduced, leading to even greater doctor attrition. This is partly due to the value placed on specialization in the Bangladesh context, and the post-graduate degree (MD/MS/FCPS/Diploma) [53]. The post-graduation examination in Bangladesh requires completion of at least two to three years of in-house training as a precondition to sit for the examination. Jobs in the public secondary and tertiary level facilities are counted as in-house training. This was not the case for the CO project given that service in primary care settings usually involves referring critical cases, versus offering senior supervision around their in-facility management. The desire for career growth driven by the focus on specialization as a measure of competence and professionalism, and fear of “career death” within the CO system, was a major factor provoking the sustained loss of project doctors to public sector. To counter this attrition, the implementation of CO projects requires much greater attention to creating career paths that emphasize professional growth and development.

The emphasis on specialization also relates to larger systemic biases against primary care in terms of remuneration and perceived value [54, 55]. Primary health care providers or “Generalists” play an important role in health prevention, maternal and child care, the treatment of uncomplicated cases and referral to specialized services, yet do not command the same wages or value as specialist providers. In Bangladesh, specialized doctors have access to improved and up-to-date medical technologies and both public and private healthcare markets, and account for a large proportion of out-of-pocket expenses which currently exceed 67% of total national healthcare expenditure [56]. A bifurcation of care has resulted, whereby poorer and marginalized populations are disproportionate users of lower cost public primary care services provided by healthcare practitioners who are relatively underpaid. By contrast, affluent patients prefer and pay for more costly services from specialists working in the urban private sector. These tendencies in the healthcare system have worked to limit the attraction of doctors to stay in PHC sector, and encourage the drift towards high prestige and remunerative specialized training as echoed in previous studies [54, 55]. In this context, the importance of creating a socially committed primary healthcare workforce is even more important, as is the use of incentives, and professional development opportunities that reward a holistic, close-to-community approach. This involves reforms in medical curricula that strengthen primary care training, drawing on experiences from industrialized and developed countries [54]. One such movement started in Brazil, where in order to slow a hemorrhage of physicians to specialized healthcare, a unified Health System was created with a strong commitment to reforms in professional medical education that emphasize primary healthcare as a gateway to specialized care [55]. Similar reforms could be considered in Bangladesh, whereby strong referral mechanisms are created and regulated to stem the overuse of specialists and promote primary healthcare as a frontline service that initiates referral.

Contributing to the lack of sensitivity to career development was the project’s situation in the MOLGRD&C, a Ministry without expertise in implementing health-related projects, and its leadership by a career bureaucrat with no health-related background. While it could be argued that doctors lack capacity and training to become good managers due to deficiencies in medical curriculum identified in the country’s latest Health Workforce Strategy 2015 [57], a clear gap is apparent that needs to be addressed. In filling higher administrative positions in health-related projects, sensitization to health sector issues is critical, as is a good grasp of management for increased efficiency and transparency.

Along with stressors related to job security and career development, fear of abuse and disrespect are common issues affecting healthcare workers even in urban areas [45, 58]. In rural Bangladesh two-thirds of healthcare providers reported having experienced disrespect from community members when they were unable to fulfill expectations regarding the provision of medicine or healthcare support [45, 59]. In this study, similar experiences were shared. Serving a largely poor and marginalized urban population with low literacy, failures in communication with community—i.e. that critical care services are not provided–likely accounted for this problem. Greater efforts to increase community awareness about service offerings, and more security during night shifts would mitigate this cause of attrition.

A final issue undermining doctor retention relates to contract design, and in particular, the adoption of the “low cost bidding” approach in the third phase of the project. By valuing financial criteria over quality considerations, the importance of investing in HRH skills, capacity and motivation was underestimated and overlooked.

Our findings point toward several areas of improvement in the design of future contracting out arrangements that address the problem of doctor retention. Given the project’s concern for population health improvement in underserved urban settings, contract design should privilege the ‘Contractee’ so that health workforce investments are prioritized even if higher project costs may result [48]. Another area needing focus are investments in collaboration and communication between donor agencies, government and NGOs around evidence-based strategies to promote doctor retention and affordable, quality healthcare [60]. In Uganda, donors led evidence generation and dissemination activities to “push” the abolition of healthcare fees in public facilities [61]. Even in Bangladesh, during the HPNSDP (Health, Population and Nutrition Sector Development, 2011–2016) preparation phase, the Planning Wing of MOH&FW utilized the technical assistance offered by donors to strategize and prioritize health sector investments [62]. As such, funding agencies like ADB have a crucial role to play by emphasizing evidence-informed changes to project design. Specifically, findings from donor-commissioned research or, midterm evaluations can inform dialogue between the PMU, NGOs and donors, help address gaps in project implementation.

The study has some limitations. As the study design was retrospective and KIs were asked to recall their experiences and recollect important events and milestones that happened twenty years ago, there is a possibility of recall bias. To minimize the impact of these biases, we drew inferences from different categories of participants and also triangulated with project documents. Several potential KIs, who were directly involved with the project, declined the interview request. In addition, the representative personnel from the lead donor, ADB were also inaccessible. The researchers communicated with past and present project consultants from that institution, and had to rely on them to capture ADB’s views.

## Conclusions

Doctors’ retention in CO arrangements for urban primary care is a challenge that requires financial and strategic investment. To this end, job security, incentives and opportunities for training and professional development, would reduce costs related to high doctor turnover and support a more efficiently functioning healthcare system, better quality of care, and ultimately, improved health outcomes. Complementing these reforms are strategies that support formal collaboration between relevant ministries, NGOs, medical universities and international donors working in the health sector, in terms of workforce planning and training for the provision of urban primary healthcare.

In the context of CO arrangements in low-resource settings, project design and the structure of bidding processes and contracts must balance a concern for minimizing administrative costs with the value of an efficient and motivated workforce. But there is no one pathway through which this can be achieved. Each setting may require context-specific solutions that work within its particular culture and health system. Bangladesh’s long history of CO points to the central, cross-cutting importance of the role of health human resources in enabling the efficient and sustainable delivery of front-line services. Recognizing the value of primary care providers, and investing in their professional development, remuneration and security are critical first steps in making CO a more viable delivery strategy in urban settings.

## Supporting information

S1 Table COREQ checklist. (DOCX)

S2 Table Interview guidelines. (DOCX)
