# Supplementary material for: Making doctors stay: Rethinking doctor retention policy in a contracted-out primary healthcare setting in urban Bangladesh
Source: PLoS One. 2022 Jan 5;17(1):e0262358. doi: 10.1371/journal.pone.0262358 (PMC8730431; doi:10.1371/journal.pone.0262358)
Supplement: S1 Table — (DOCX) [file pone.0262358.s001.docx]

**Supplementary Table 1. COREQ checklist (1)**

For

**“Making Doctors Stay: Rethinking Doctor Retention Policy in a Contracted-Out Primary Healthcare Setting in Urban Bangladesh”**

| **Topic** | **Item No.** | **Guide Questions/Description** | **Details/ reported on page no.** |
| --- | --- | --- | --- |
| **Domain 1: Research team and reﬂexivity** | | | |
| *Personal characteristics* | | | |
| Interviewer/facilitator | 1 | Which author/s conducted the interview or focus group? | Farzana Bashar  Rubana Islam  Shaan Muberra Khan  Shahed Hossain  Adel A. S. Sikder  Sifat S Yusuf  Alayne M Adams  Page no. 1 |
| Credentials | 2 | What were the researcher’s credentials? E.g. PhD, MD | Farzana Bashar (FB), MBBS, MPH  Rubana Islam (RI), MBBS, MPH, PhD  Shaan Muberra Khan (SMK), BSc, MPH  Shahed Hossain (SH), MBBS, PhD  Adel A.S Sikder (AASS), MBBS, MPH  Sifat S Yusuf (SSY), BSc, MPH  Alayne M Adams (AMA), PhD  Non-author data transcribers held Master’s degree. |
| Occupation | 3 | What was their occupation at the time of the study? | FB, RI, SMK, SH, AASS, SSY and AMA were full time researchers with extensive experience in the field of health systems research. All of them worked at International Centre for Diarrhoeal Disease Research, Bangladesh (icddr,b) when the study was conducted. RI was the Principal Investigator (PI) of the project, while AMA and SH were the Co-PIs. FB, SMK and AASS were the major contributors in project implementation, data collection, management, analysis and reporting under direct supervision of RI, SH and AMA. SSY had participated substantially in data collection, management and analysis.  Non-author data transcribers were full-time researchers.  Page no. 1 |
| Gender | 4 | Was the researcher male or female? | Farzana Bashar (FB), Female  Rubana Islam (RI), Female  Shaan Muberra Khan (SMK), Female  Shahed Hossain (SH), Male  Adel A.S Sikder (AASS), Male  Sifat S Yusuf (SSY), Female  Alayne M Adams (AMA), Female |
| Experience and training | 5 | What experience or training did the researcher have? | MPH: FB, SMK, AASS, SSY  PhD: SH, AMA,RI |
| *Relationship with participants* | | | |
| Relationship established | 6 | Was a relationship established prior to study commencement? | No, no relationship established between researchers and research participants. However, some of the researchers had long standing professional relationships of several of the participants. |
|  | 7 | What did the participants know about the researcher? e.g. personal goals, reasons for doing the research | Participants were informed about the objectives of the study and how the researchers felt the results could help to improve the implementation of similar health programs. |
| Interviewer characteristics | 8 | What characteristics were reported about the inter viewer/facilitator? e.g. Bias, assumptions, reasons and interests in the research topic | Participants were informed about the objectives of the study and how the researchers felt the results could help to improve the implementation of similar health programs. |
| **Domain 2: Study design** | | | |
| *Theoretical framework* | | | |
| Methodological orientation and Theory | 9 | What methodological orientation was stated to underpin the study? e.g. grounded theory, discourse analysis, ethnography, phenomenology,  content analysis | In our study, we aimed to explore the factors influencing doctor’s retention both in managerial and service provision level in the contracted-out setting. We used two theoretical frameworks, the Health Policy Triangle of Walt & Gibson (1994) and the conceptual framework for monitoring and evaluating primary health care contracting-out interventions developed by Liu et al. (2004) to guide our data collection and analysis.  Page no. 7 |
| *Participant selection* | | | |
| Sampling | 10 | How were participants selected? e.g. purposive, convenience,  consecutive, snowball | The participants were purposively selected to allow the maximum variation. Snowball sampling was also followed to ensure richness of data.  Page no. 7 |
| Method of approach | 11 | How were participants approached? e.g. face-to-face, telephone, mail,  Email | All key informant interviews were face-to-face.  Page no. 8 |
| Sample size | 12 | How many participants were in the study? | Detailed information on study participants in Table 1.  Page no. 8 |
| Non-participation | 13 | How many people refused to participate or dropped out? Reasons? | Several participants at service provider, NGO and donor level did not respond to our request. Reasons were not disclosed. |
| *Setting* | | | |
| Setting of data collection | 14 | Where was the data collected? e.g. home, clinic, workplace | Data was collected at either workplace or, residence of participants, as preferred by them. |
| Presence of non-  participants | 15 | Was anyone else present besides the participants and researchers? | No, no non-participant was present besides the participants and researchers/data collectors for reasons of confidentiality. |
| Description of sample | 16 | What are the important characteristics of the sample? e.g. demographic  data, date | Participants were donor representatives, government’s ministry officials, project personnel, NGO representatives and doctors (clinic medical officers) working at NGOs.  Page no. 8 |
| *Data collection* | | | |
| Interview guide | 17 | Were questions, prompts, guides provided by the authors? Was it pilot tested? | Yes, all data collection tool were field tested by the researchers.  Page no. 7 |
| Repeat interviews | 18 | Were repeat inter views carried out? If yes, how many? | None. |
| Audio/visual recording | 19 | Did the research use audio or visual recording to collect the data? | Key informant interviews were recorded using tape recorders where the participants consented to record the interview; if denied to record detailed notes were taken Recordings of interviews were transcribed in verbatim.  Page no. 8, 9 |
| Field notes | 20 | Were ﬁeld notes made during and/or after the interview or focus group? | The researchers took field notes while conducting the interviews. At least two researchers were sent for data collection, one facilitated the interview/ discussion and another took notes. |
| Duration | 21 | What was the duration of the inter views or focus group? | Duration of key informant interviews was varied between 30 to 90 minutes.  Page no. 8 |
| Data saturation | 22 | Was data saturation discussed? | Yes, data saturation was discussed during the research process. We stopped data collection at the point of data saturation and point data of redundancy. |
| Transcripts returned | 23 | Were transcripts returned to participants for comment and/or correction? | Transcripts were not returned. However, findings were shared with some of the participants at a stakeholder meeting for member checking.  Page no. 9 |
| **Domain 3: analysis and ﬁndings** | | | |
| *Data analysis* | | | |
| Number of data coders | 24 | How many data coders coded the data? | FB, SMK, AASS, and SSY coded the data under guidance of RI. |
| Description of the coding  tree | 25 | Did authors provide a description of the coding tree? | *a priori* codebook was developed and followed. Codes were not discussed, but available upon request.  Page no. 9 |
| Derivation of themes | 26 | Were themes identiﬁed in advance or derived from the data? | Themes were emerged/identified from the data.  Page no. 9 |
| Software | 27 | What software, if applicable, was used to manage the data? | Qualitative data analysis software, ATLAS.ti used to manage the data.  Page no. 9 |
| Participant checking | 28 | Did participants provide feedback on the ﬁndings? | Yes.  Page no. 9 |
| *Reporting* | | | |
| Quotations presented | 29 | Were participant quotations presented to illustrate the themes/ﬁndings?  Was each quotation identiﬁed? e.g. participant number | Yes.  Page no. 10-16 |
| Data and ﬁndings consistent | 30 | Was there consistency between the data presented and the ﬁndings? | Yes.  Page no. 10-16 |
| Clarity of major themes | 31 | Were major themes clearly presented in the ﬁndings? | Yes, presented in the findings.  Page no. 10-16 |
| Clarity of minor themes | 32 | Is there a description of diverse cases or discussion of minor themes? | Yes.  Page no. 10-16 |

**Developed from:** Tong A, Sainsbury P, Craig J. Consolidated criteria for reporting qualitative research (COREQ): a 32-item checklist for interviews and focus groups. *International Journal for Quality in Health Care*. 2007. Volume 19, Number 6: pp. 349 –357.

1. Tong A, Sainsbury P, Craig J. Consolidated criteria for reporting qualitative research (COREQ): a 32-item checklist for interviews and focus groups. International Journal for Quality in Health Care. 2007;19(6):349-57.
