# Supplementary material for: Making doctors stay: Rethinking doctor retention policy in a contracted-out primary healthcare setting in urban Bangladesh
Source: PLoS One. 2022 Jan 5;17(1):e0262358. doi: 10.1371/journal.pone.0262358 (PMC8730431; doi:10.1371/journal.pone.0262358)
Supplement: S2 Table — (DOCX) [file pone.0262358.s002.docx]

**Supplementary Table 2. Interview guidelines**

**Key Informant Interview Guide- Donor/Contract designers/Ministry of Health & Family Welfare**

**Note:**

1. Start by introducing yourself, the purpose and the expected time (1 hour). If agreeable to interview, proceed to the consent form
2. Request to record interview and make sure the tape recorder is switched on to the start of the interview. If denied, make note.
3. **Make note of the phase of the UPHCSDP being interviewed for- 1/2/3**

**Socio-demographic information**

1. Organization
2. Current Position
3. Organization and Position during the project period.

**Ice breaker**:

1. Since when you are/were involved in this project? Why did you think that contracting out can be an effective means to address urban PHC needs in this context?
2. How has UPHCP helped in improving the primary health care situation in Urban Bangladesh?
3. From your experience what are strengths and weaknesses of the project?

Section 1: Project history

1. What were the reasons to start Contracting out (CO) Health Services in Bangladesh?
2. How was the project planned? Who was involved?
3. What were the series of major events that happened during contract development and negotiations with the donors/GoB/NGO?
4. How has the design and plan for CO in BD changed over time? Were they reflected in the agreement?
5. Tell us some highlights/milestones of the changes. When did they occur?
6. In what ways would you say is the project different/improved than in the last 2 phases?

Section 2: Actors

1. Who were the major actors involved in the contracting process (Design and implementation?
2. What was the role of different actors?
3. What would you say were their positions and perspectives toward CO?
4. Please comment about their communication and collaboration.
5. Whose support would most importantly change the fate of CO in BD?

Section 3: Context

1. Have you/or the bank/donor group? been involved with any other similar project in any other country? If yes, what is your experience there and how did it differ from UPHCP?
2. In your experience what are the main external factors that have influenced the evolution of CO in BD?

Probe:

- *International and national health goals , Influence form outside the health sector, Donor interest, Constraints in 6 health system building blocks (HR availability/turnover, service quality, coverage, technology, governance, financing, information system), Judicial system*

1. Which factors were supportive of CO mechanism and implementation?
2. Which were not?
3. Which factors could have been handled differently to facilitate CO better?

Section 4: Content

1. What were the major issues that were considered during the development of the contracts (ask separately for each phase)?

Probe:

- *Inter-ministerial cooperation, Donor retraction, Fund disbursement, Provider payment, New health focus/concern, Results from evaluation*

1. What were the strengths of the contract? Weaknesses?
2. What factors affected the changes in the design of the contract?
3. Were the changes made in the contract in the subsequent phases relevant to the difficulties experienced?
4. What more or different would you want to see in the agreement?

Section 5: Process

1. Were the changes helpful in improving the implementation of CO? How?
2. What factors hindered the implementation/delivering services as planned/required? Which areas if work were affected most?
3. What do you think about involvement of multiple donor organization in this project? How did this influence the whole contracting out mechanism?
4. What happened in the transition phase between two contracts?
5. Do you think CO should be integrated in Urban Health Policy/Strategy in BD?
6. If yes, what could facilitate this process? If no, why not?

Section 6: NGO performance

1. What did you think were the main differences between the providers agencies contracted? Was it different over the three phases?
2. Overall how would you rate the performance of the providers?
3. In what aspects did their performance vary across providers?

Probe:

- *Access, quality, equity, efficiency, input (human resource, drug, equipment’s/supplies), and output management (program planning, administration, and finance, hiring and procurement practices, and client satisfaction systems)*

1. If their performances varied what could have been the main reasons?

Probe:

- *The contract: Contractual relationships, type of services and their contractibility (Services with clear or unclear level of need, Technical complexity of contracted services), contract formality, contract duration, provider selection, specification of performance requirements, specifications of output and outcomes, provider payment mechanisms*
- *Contractor: Type of purchaser, managerial and financial capacity( previous experience in contracting management, ability to provide technical assistance in designing and managing contracting-out initiative, financing sufficiency for contracted services, stability of financing over years), performance monitoring*
- *Provider: type, capacity (experience in contracting with government, financial health prior to contracting, capacity for provision of contracted services at the right location to the right people prior to contracting, presence of information systems allowing for M&E and performance management, level of entrepreneurship of upper management), competition (other private providers)*
- *External factors: Regulatory and legal environment, banking systems, organization of health service delivery, stance and efficiency of public providers, level of efficiency of public providers, level of corruption*

Finish the Interview by thanking the participant.

**Interview guidelines**

**Key Informant Interview Guide- Project Management Unit/NGO Heads**

**Note:**

1. Start by introducing yourself, the purpose and the expected time (1 hour). If agreeable to interview, proceed to the consent form
2. Request to record interview and make sure the tape recorder is switched on to the start of the interview. If denied, make notes
3. **Make note of the phase of the UPHCSDP being interviewed for- 1/2/3**

**Socio-demographic information**

1. Organization
2. Current Position
3. Organization and Position during the project period.

**Ice breaker**:

1. Since when you are/were involved in this project? Why did you think that contracting out can be an effective means to address urban PHC needs in this context?
2. How has UPHCP helped in improving the primary health care situation in Urban Bangladesh?
3. From your experience what are strengths and weaknesses of the project?

Section 1: Project history

1. Why do you think Contracting out (CO) Health Services started in Bangladesh?
2. How was the project planned? Who was involved?
3. What were the series of major events that happened during contract development and negotiations with the donors/GoB/NGO?
4. How has the design and plan for CO in BD changed over time? Were they reflected in the agreement?
5. Tell us some highlights/milestones of the changes. When did they occur?
6. In what ways would you say is the project different/improved than in the last 2 phases?

Section 2: Actors

1. Who were the major actors involved in the contracting process (design and implementation?
2. What was the role of different actors?
3. What would you say were their positions and perspectives toward CO?
4. Whose support would most importantly change the fate of CO in BD?
5. Please comment about their communication and collaboration.

Section 3: Context

1. In your experience what are the main external factors that have influenced the evolution of CO in BD?

Probe:

- *International and national health goals , Influence form outside the health sector, Donor interest ,Constraints in 6 health system building blocks (HR availability/turnover, service quality, coverage, technology, governance, financing, information system), Judicial system*

1. Which factors were supportive of CO mechanism and implementation?
2. Which were not?
3. Which factors could have been handled differently to facilitate CO better?

Section 4: Content

1. What were the major issues that were considered during the development of the contracts (ask separately for each phase)?

Probe:

- *Inter-ministerial cooperation, Donor retraction, Fund disbursement, Provider payment, New health focus/concern, Results from evaluation*

1. What were the strengths of the contract? Weaknesses?
2. What factors affected the changes in the design of the contract?
3. Were the changes made in the contract in subsequent phases relevant to the difficulties experienced?
4. What more or different would you want to see in the agreement?
5. Why did you work for the project (for PMu)?/ Why did you join the agreement (For NGO)?
6. If you no longer work with the project, what made you leave?
7. Which clauses in the contract did you find agreeable? Which were not agreeable?
8. Were they easy to meet? Why not?
9. Has your involvement changed in any way in the 3 phases? If so how?
10. What more or different would you want to see in the agreement?

Section 5: Process

1. What factors hindered the implementation/delivering services as planned/required? Which areas if work were affected most?
2. Were the changes helpful in improving the implementation of CO? How?
3. What do you think about involvement of multiple donor organization in this project? How did this influence the whole contracting out mechanism?
4. What happened in the transition phase between two contracts?
5. Do you think CO should be integrated in Urban Health Policy/Strategy in BD?
6. What could facilitate this process?

Section 6: NGO performance

1. What did you think were the main differences between the providers agencies contracted? Was it different over the three phases?
2. In what aspects did their performance vary?

Probe:

- *Access, quality, equity, efficiency, input (human resource, drug, equipment’s/supplies), and output management (program planning, administration, and finance, hiring and procurement practices, and client satisfaction systems)*

1. If their performances varied what were the main reasons?

Probe:

- *The contract: Contractual relationships, type of services and their contractibility (Services with clear or unclear level of need, Technical complexity of contracted services), contract formality, contract duration, provider selection, specification of performance requirements, specifications of output and outcomes, provider payment mechanisms*
- *Contractor: Type of purchaser, managerial and financial capacity( previous experience in contracting management, ability to provide technical assistance in designing and managing contracting-out initiative, financing sufficiency for contracted services, stability of financing over years), performance monitoring*
- *Provider: type, capacity (experience in contracting with government, financial health prior to contracting, capacity for provision of contracted services at the right location to the right people prior to contracting, presence of information systems allowing for M&E and performance management, level of entrepreneurship of upper management), competition (other private providers)*
- *External factors: Regulatory and legal environment, banking systems, organization of health service delivery, stance and efficiency of public providers, level of efficiency of public providers, level of corruption*

Finish the Interview by thanking the participant.

**Interview guidelines**

**Key Informant Interview Guide- NGO Managers/Clinic Medical Officers**

**Note:**

1. Start by introducing yourself, the purpose and the expected time (1 hour). If agreeable to interview, proceed to the consent form
2. Request to record interview and make sure the tape recorder is switched on to the start of the interview. If denied, make note

**Socio-demographic information**

1. Organization
2. Current Position
3. Organization and Position during the project period.

**Ice breaker**:

1. Since when you are/were involved in this project? Why did you think that contracting out can be an effective means to address urban PHC needs in this context?
2. How has UPHCP helped in improving the primary health care situation in Urban Bangladesh?
3. From your experience what are strengths and weaknesses of the project?
4. Do you think CO should be integrated in Urban Health Policy/Strategy in BD? Why?

Section 1 NGO performance

1. What did you think were the main differences between the providers agencies contracted? Was it different over the three phases?
2. In what aspects did their performance vary?

Probe:

- *Access, quality, equity, efficiency, input (human resource, drug, equipment’s/supplies), and output management (program planning, administration, and finance, hiring and procurement practices, and client satisfaction systems)*

1. If their performances varied, what were the main reasons?

Probe:

- *The contract: Contractual relationships, type of services and their contractibility (Services with clear or unclear level of need, Technical complexity of contracted services), contract formality, contract duration, provider selection, specification of performance requirements, specifications of output and outcomes, provider payment mechanisms*
- *Contractor: Type of purchaser, managerial and financial capacity( previous experience in contracting management, ability to provide technical assistance in designing and managing contracting-out initiative, financing sufficiency for contracted services, stability of financing over years), performance monitoring*
- *Provider: type, capacity (experience in contracting with government, financial health prior to contracting, capacity for provision of contracted services at the right location to the right people prior to contracting, presence of information systems allowing for M&E and performance management, level of entrepreneurship of upper management), competition (other private providers)*
- *External factors: Regulatory and legal environment, banking systems, organization of health service delivery, stance and efficiency of public providers, level of efficiency of public providers, level of corruption*

1. What factors hindered the delivering services as planned/required? Which areas if work were affected most?
2. What were the most challenging aspects of managing service provision in the clinics?
3. What/who helped to overcome those challenges? How?

Finish the Interview by thanking the participant.
